# A PPy/ZnO functional interlayer to enhance electrochemical performance of lithium/sulfur batteries

**DOI:** 10.1186/s11671-018-2724-x

**Published:** 2018-10-03

**Authors:** Fuxing Yin, Jun Ren, Yongguang Zhang, Taizhe Tan, Zhihong Chen

**Affiliations:** 1 School of Materials Science and Engineering, Research Institute for Energy Equipment Materials, Hebei University of Technology, Tianjin, 300130 China; 2 Synergy Innovation Institute of GDUT, Heyuan, Guangdong Province China; 3 Shenyang Institute of Automation, Chinese Academy of Sciences, Guangzhou, 511458 China

**Keywords:** Lithium/sulfur battery, PPy/ZnO composite, Interlayer, Electrochemical performance

## Abstract

To enhance the electrochemical performance of the lithium/sulfur batteries, a novel interlayer was prepared by coating the slurry of PPy/ZnO composite onto the surface of a separator. Owing to a three-dimensional hierarchical network structure, PPy/ZnO composite serves as a polysulfide diffusion absorbent that can intercept the migrating soluble polysulfides to enhance the electrochemical performance of the Li/S batteries. The specific capacity of the cell with PPy/ZnO interlayer remained at 579 mAh g^−1^ after 100 cycles at 0.2 C. This interlayer can provide novel avenues for the commercial applications of Li/S batteries.

**Electronic supplementary material:**

The online version of this article (10.1186/s11671-018-2724-x) contains supplementary material, which is available to authorized users.

## Background

With the increasing development of the portable electronic devices and negative impact of conventional energy systems, the development of high-performing, light-weight energy storage systems has attracted significant attention. Lithium/sulfur (Li/S) batteries are regarded as a likely alternative because of their high energy density of 2600 Wh kg^−1^ and theoretical capacity of 1672 mAh g^−1^ [1, 2]. However, their performance is limited by low-conductivity of active material and the polysulfide shuttle mechanism [3–5].

In the last few decades, several strategies have been tested to overcome these challenges, such as encapsulation of the active material within nanostructure, development of novel electrolytes, and binder modification [6–9]. The insertion of an interlayer between cathode and separator can significantly improve the capacity retention by trapping polysulfides [10–12]. However, a low adsorption capacity of carbon for polysulfides still restricts the cycling performance of Li/S batteries. Studies demonstrate that PPy is a proton-doped type of conductive polymer. This type of conductive polymer can adsorb polysulfides by H bonds. Therefore, PPy is suitable for fabrication as an interlayer to suppress the shuttling of polysulfides [13]. Also, the polar metal oxides can form chemical bonds with polysulfides to decrease the loss of active materials [14–16]. Yu et al. [17] have reported that ZnO coating could effectively confine polysulfides during cycling. However, these metal oxides reduce the utilization of sulfur due to their low electrical conductivity.

After comprehensive considerations, in order to realize the high performance of Li/S batteries, a novel interlayer composed of polypyrrole (PPy) and ZnO nanoparticle has been fabricated. The cross-linked PPy nanofibers formed a three-dimensional hierarchical network structure in the composite which was uniformly coated by ZnO nanoparticles. We hypothesized that the interlayer with special morphology would provide both the chemical and physical restraints to hinder the diffusion of polysulfides and protect the active material to suppress “shuttle effect.” The combination of PPy and ZnO not only enhances the ability of the interlayer to capture polysulfide but also avoids the defect of poor conductivity of the ZnO-only interlayer. Moreover, such a 3D structure can offer better electronic pathways and reduce the electrochemical polarization. To prove the effectiveness of such an interlayer in enhancing the performance of Li/S batteries, we uniformly coated PPy/ZnO composite onto the surface of a separator as an interlayer.

## Methods

### Preparation of PPy/ZnO interlayer

PPy nanofiber network was synthesized as previously reported [18]. The as-prepared PPy (0.2 g) was added in Zn (CH_3_COO)_2_•2H_2_O methanol solution (4 mM, 30 mL) under magnetic stirring. Then, potassium hydroxide (KOH) methanol solution (0.3 M, 10 mL) was added and the mixture was transferred into an oil bath at 60 C under continuous stirring. Finally, the PPy/ZnO composite was obtained by centrifugation. The mixed slurry of PPy/ZnO composite, Ketjen Black (EC 300 J), and polyvinylidene fluoride (PVDF) (80:10:10 in weight ratio) was coated uniformly onto the surface of the separator (Celgard 2300) to fabricate interlayer.

### Preparation of S-cathode

Sulfur (Sigma-Aldrich, ~ 100 mesh particle size) and graphene were thoroughly mixed in the weight ratio of 2:1 and then heated at 155 C for 12 h under argon atmosphere. The sulfur cathode was fabricated by mixing S/graphene composite, Ketjen Black, and PVDF (80:10:10 in weight ratio). The slurry was smeared on carbon-coated aluminum foil. After drying at 60 C for 12 h, the cathode was obtained by punching using a disk with 14 nm-in-diameter. The sulfur loading is approximately 1.3 mg cm^−2^.

### Material characterization

The samples were characterized by field emission scanning electron microscopy (FE-SEM, Leo-1530), transmission electron microscopy (TEM, JEM-2100F), X-ray diffractometer (XRD, Smart Lab), Fourier-transform infrared spectroscopy (FTIR, TENSOR 27), and X-ray photoelectron spectroscopy (XPS, Thermo ESCALAB 250Xi).

### Electrochemical measurements

The half-cell assembly was developed in a glove-box filled with Ar (99.9995% purity). Lithium foil was applied as the anode and a mixed solution of 1 M LiN (CF_3_SO_2_)_2_ (LiTFSI) with 0.1 M LiNO_3_ dissolved in a solution of 1,3-dioxolane (DOL) and 1,2-dimethoxyethane (DME) (*v*/*v* 10:10) was prepared as electrolyte. The amount of electrolyte is around 30 μL. The as-made half-cell was tested in the voltage range of 1.7–2.8 V using battery testing station (Neware). The VersaSTAT 4 electrochemical workstation was performed to test cyclic voltammetry (CV, 1.7–2.8 V) and electrochemical impedance spectroscopy (EIS, 10^−2^–10^5^ Hz). The scan rate of CV was 0.1 mV s^−1^.

## Results and discussion

**Fig. 1:**
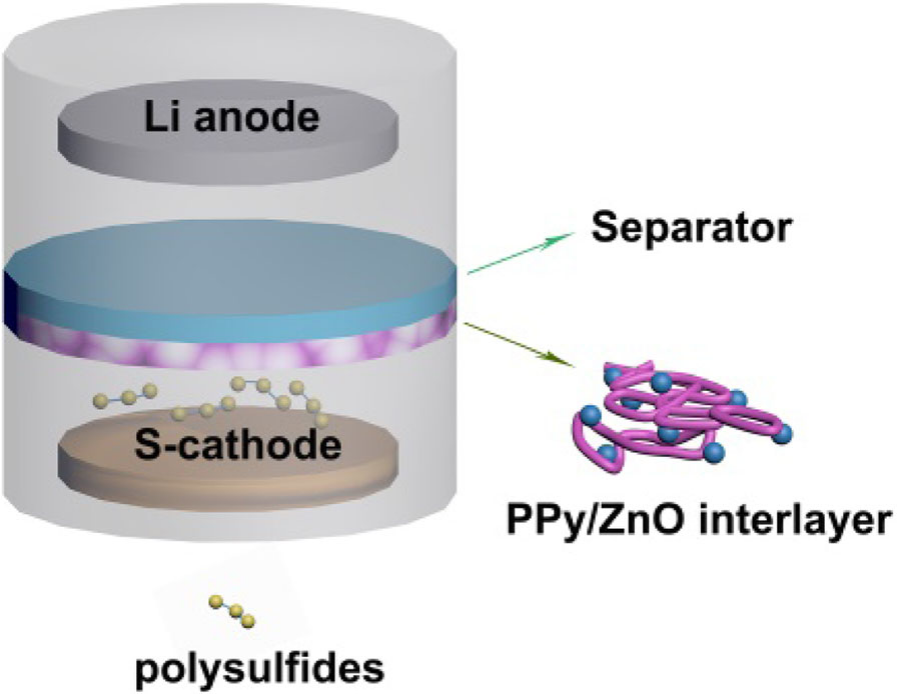
A schematic of the cell with PPy/ZnO interlayer

The structure of the cell with PPy/ZnO interlayer is shown in Fig. 1. The PPy/ZnO composite was coated uniformly onto the surface of the separator to fabricate interlayer to trap polysulfides.

**Fig. 2:**
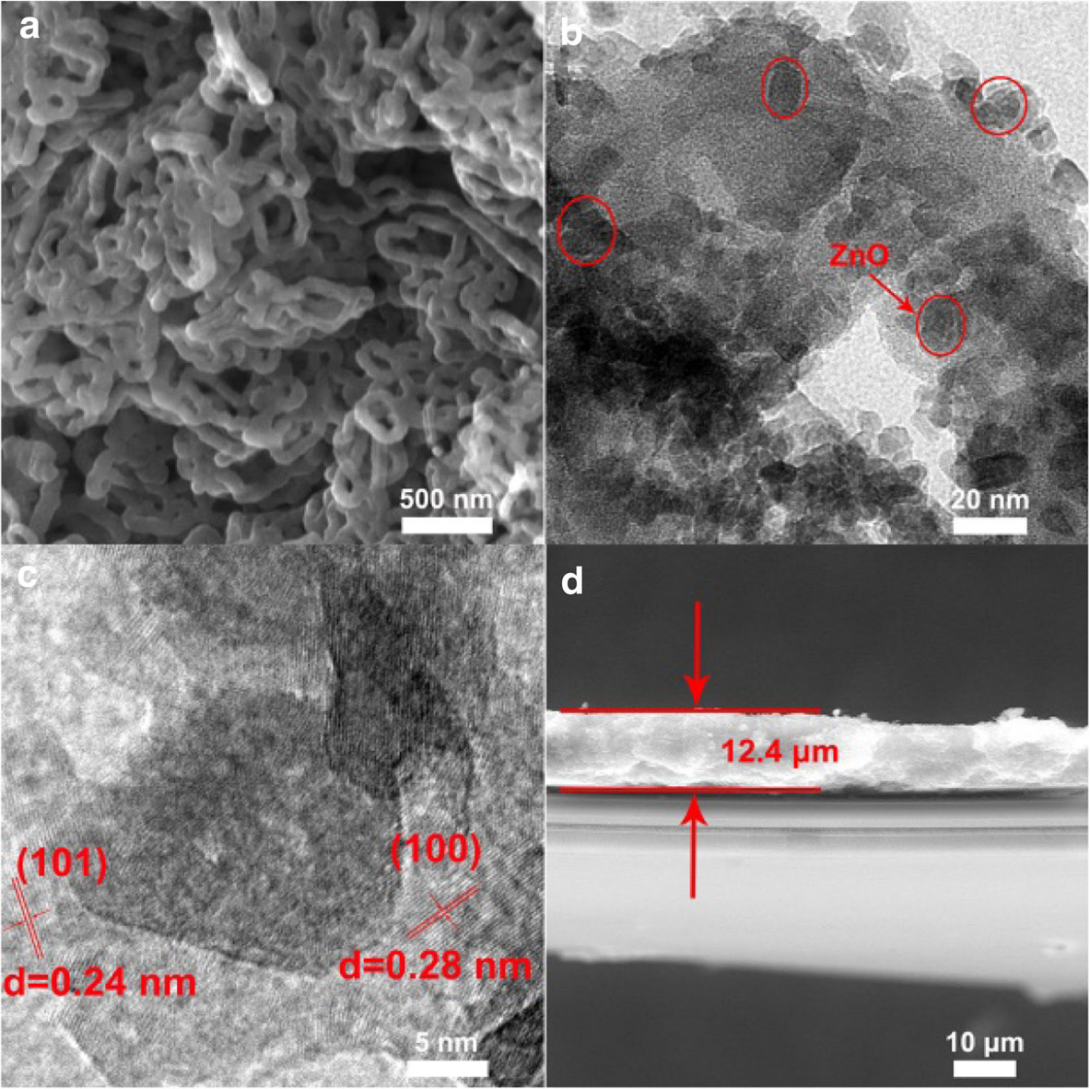
**a** SEM image of PPy/ZnO composite. **b–c** TEM images of PPy/ZnO composite at different magnifications. **d** cross-sectional SEM image of PPy/ZnO composite-coated separator

SEM and TEM were employed to investigate the morphology and size of PPy/ZnO composite. As seen in Fig. 2a, PPy/ZnO composite was obtained with a three-dimensional hierarchical network structure made up of cross-linked nanofibers. The ZnO nanoparticles were clearly present in the composite (Fig. 2c) and grew uniformly on the surface of PPy nanofiber (Fig. 2b). The diameter of PPy nanofiber and ZnO nanoparticle was about ~ 80 nm and ~ 15 nm, respectively. Clear lattice fringes can be observed in Fig. 2c indicating the presence of ZnO with the various lattice spacing of 0.24 and 0.28 nm, which can be assigned to (101) plane and (100) plane, respectively. The thickness of PPy/ZnO interlayer was estimated to be around 12.4 nm from the cross-sectional images via SEM (Fig. 2d).

**Fig. 3:**
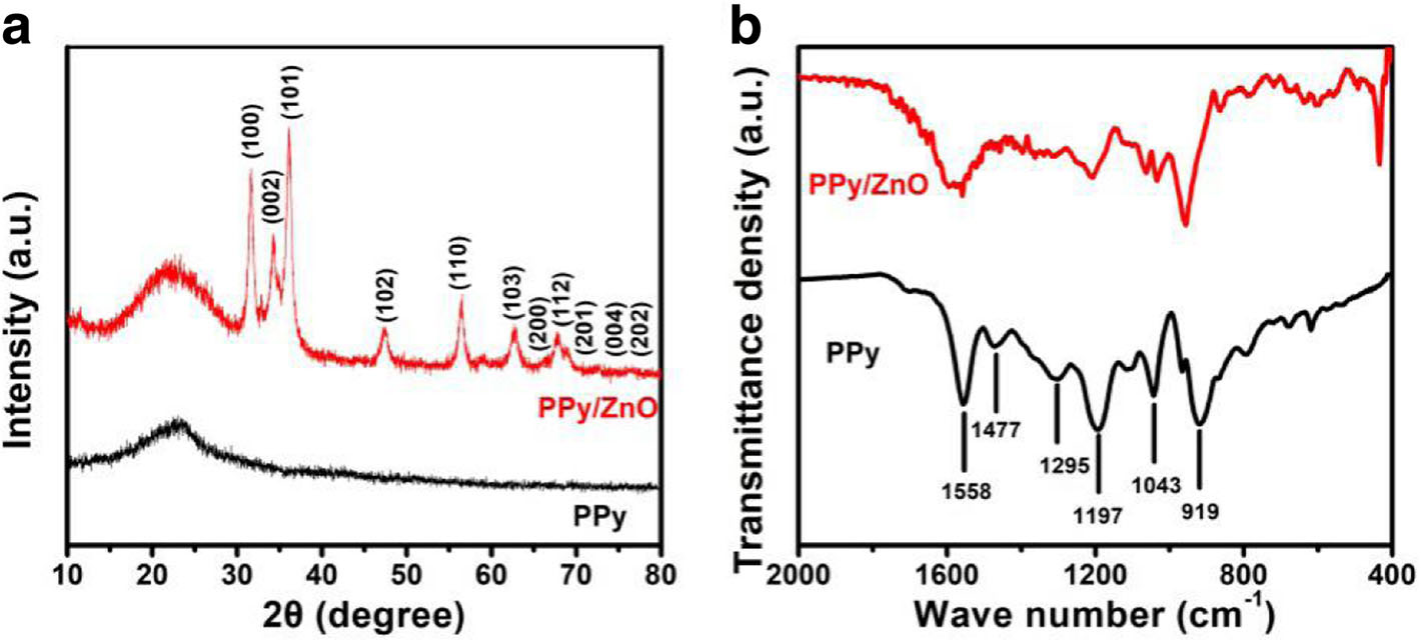
**a** XRD patterns of the PPy and PPy/ZnO composite and **b** FTIR spectra of PPy and PPy/ZnO composite

Figure 3a displays the XRD patterns of PPy and PPy/ZnO composite. We can observe a diffraction peak at about 24^°^, a characteristic of PPy, which corresponds to a typical amorphous character [19]. The PPy/ZnO composite presented the typical diffraction peaks of the hexagonal wurtzite structure of ZnO (JCPDS card No. 36-1451). FTIR spectra of PPy and PPy/ZnO composite recorded in the range of 400–2000 cm^−1^ are presented in Fig. 3b. The characteristic bands of PPy at 1533 and 1456 cm^−1^ were attributed to the pyrrole ring fundamental vibrations. The bands at around 1033, 1164, and 1286 cm^−1^ were assigned to N-H, C-N-C, and = C-H, respectively [20]. In the spectrum of PPy/ZnO composite, the peak at 437 cm^−1^ was attributed to Zn-O stretching vibration of ZnO.

**Fig. 4:**
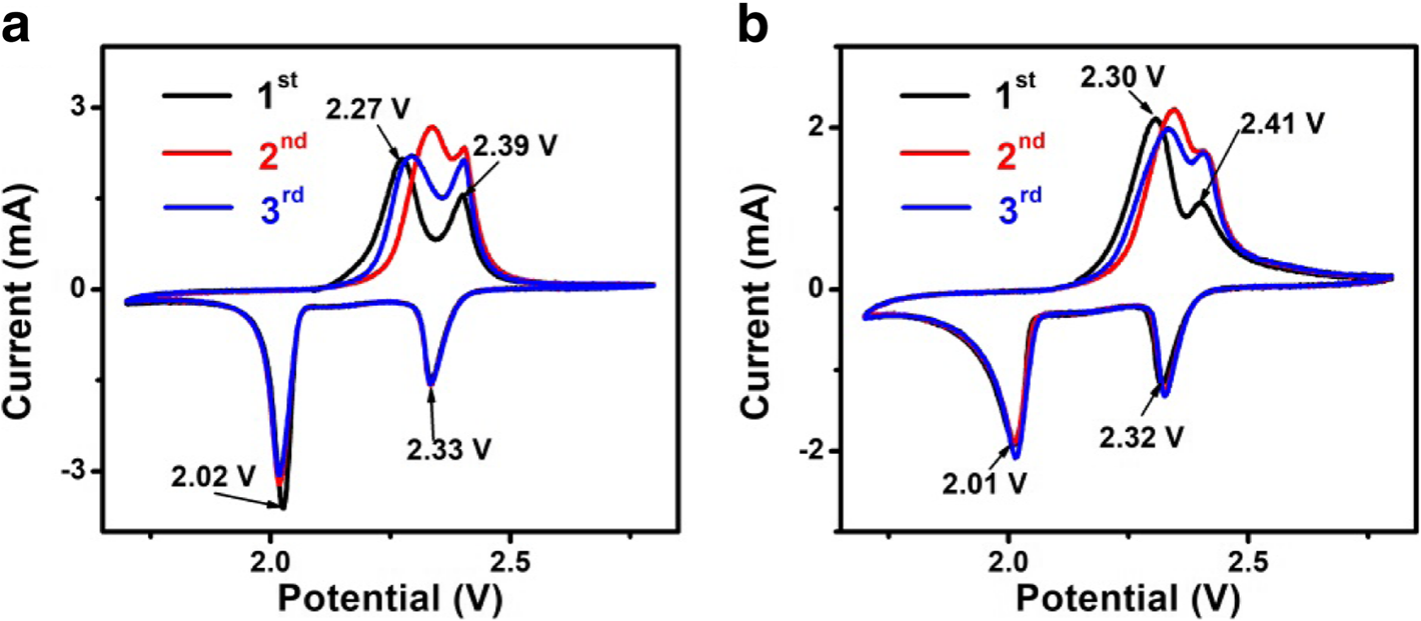
CV profiles of cells with PPy/ZnO interlayer (**a**) and without PPy/ZnO interlayer (**b**)

The electrochemical performance of the as-prepared cell with PPy/ZnO interlayer and without PPy/ZnO interlayer is shown in Fig. 4. All the CV curves show two reduction peaks and two oxidation peaks. Two reduction peaks are related to the active material forming higher order polysulfides (Li_2_S_n_, 4 ≤ *n* ≤ 8) and a further reduction to form lower order polysulfides (Li_2_S_2_/Li_2_S), respectively [21–23]. Two oxidation peaks correspond to the conversion of Li_2_S_2_/Li_2_S into higher order polysulfides further to S [24]. By comparing the peak positions, the insertion of PPy/ZnO interlayer can reduce the kinetic barrier for the redox reaction of active material and possibly lower the electrochemical polarization [25].

**Fig. 5:**
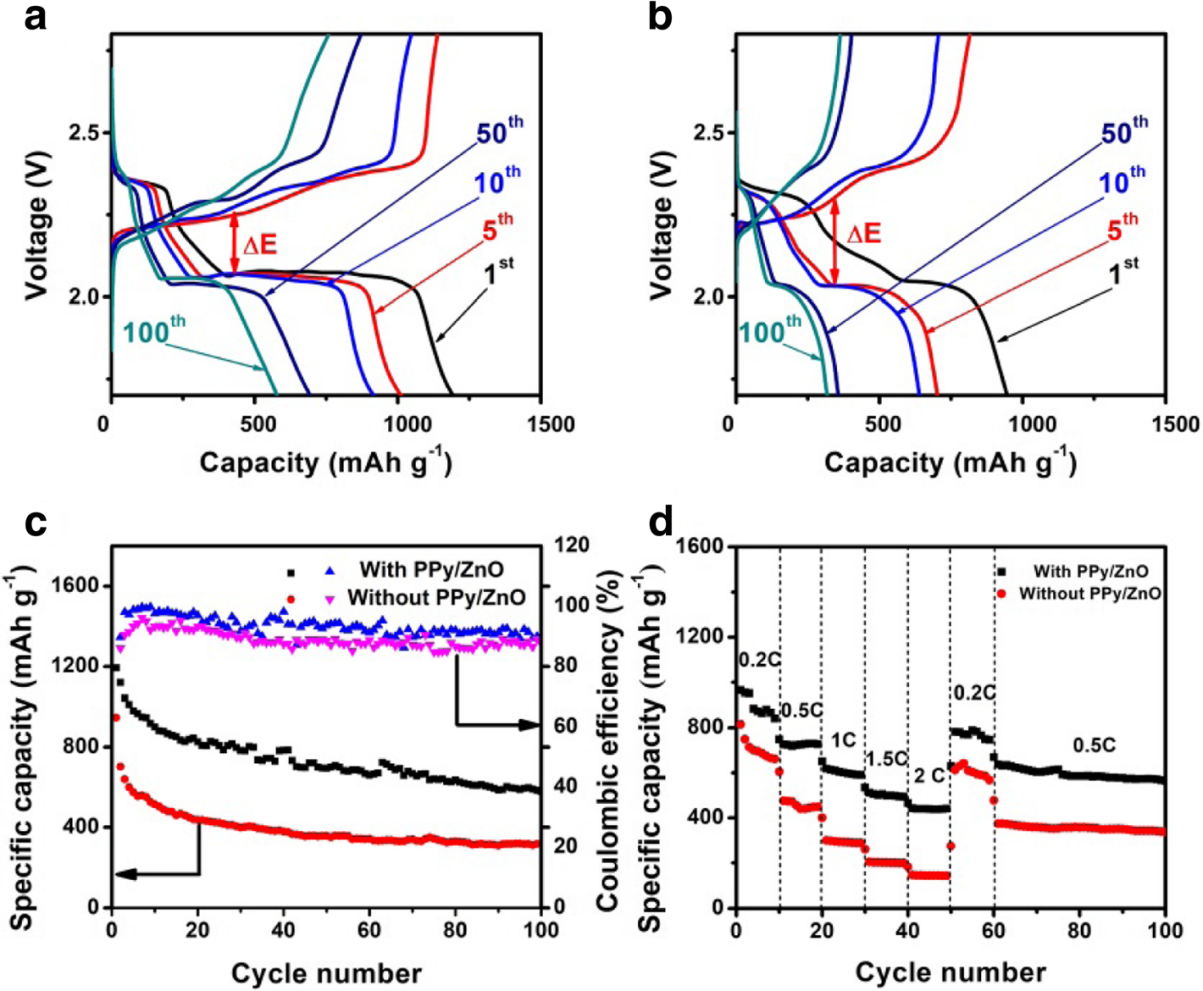
Galvanostatic charge/discharge profiles of cells with PPy/ZnO interlayer (**a**), without PPy/ZnO interlayer (**b**) at 0.2 C; the cycling performance at 0.2 C (**c**) and rate performance (**d**) of cells with PPy/ZnO interlayer and without PPy/ZnO interlayer

Galvanostatic charge/discharge voltage profiles were measured at 0.2 C to investigate the cycle performances of the as-prepared Li/S batteries. Fig. 5a, b present the charge/discharge profiles in the 1st, 5th, 10th, 50th, and 100th cycles. These profiles are in good agreement with the CV measurements. Compared with the cell without PPy/ZnO interlayer, the cell with PPy/ZnO interlayer has lower difference between the long lower discharge plateau and charge plateau. In other words, the cell with PPy/ZnO interlayer had a lower ΔE value than the one without PPy/ZnO interlayer. These results are consistent with the CV curves peaks and further indicate that PPy/ZnO interlayer can reduce the polarization. Also, the cell with PPy/ZnO interlayer revealed more stable discharge plateaus than that without PPy/ZnO interlayer.

The cell with PPy/ZnO interlayer exhibited an initial capacity of 1194 mAh g^−1^ and still delivered a discharge capacity of 579 mAh g^−1^ at 0.2 C after 100 cycles (Fig. 5c). In contrast, the capacity of Li/S batteries without PPy/ZnO interlayer was reduced to 318 mAh g^−1^ after 100 cycles, revealing serious capacity fading (Additional file 1). Hence, by inserting the interlayer, the initial discharge capacity is significantly increased and the capacity decay rate is significantly reduced. These results further illustrate that the polysulfides are absorbed by PPy/ZnO interlayer instead of diffusing to the anode and the interlayer can remarkably promote the reuse of active materials [26].

The as-prepared Li/S batteries with or without PPy/ZnO interlayer were also tested at varying current densities between 0.2 C and 2 C. The discharge capacities of the cell with PPy/ZnO interlayer were approximately 951, 718, 609, 501, and 404 mAh g^−1^ at 0.2 C, 0.5 C, 1 C, 1.5 C, and 2 C, respectively (Fig. 5d). A stable capacity of 770 mAh g^−1^ resumed when the current rate was returned back to 0.2 C. The cell without PPy/ZnO interlayer delivered 714 mAh g^−1^, 472 mAh g^−1^, 295 mAh g^−1^, 202 mAh g^−1^, and 144 mAh g^−1^ at 0.2 C, 0.5 C, 1 C, 1.5 C, and 2 C, respectively. When the current rate was switched back to 0.5 C, the reversible capacity (564 mAh g^−1^) of the cell with PPy/ZnO interlayer after 40 cycles was higher than that without PPy/ZnO interlayer. These results further validate the excellent cycling stability of the cell with PPy/ZnO interlayer. The possible reason for the phenomenon could be that PPy/ZnO composite as functional interlayer with ultrahigh adsorption capability can limit the dissolution and diffusion of polysulfides to enhance cycling stability [23].

**Fig. 6:**
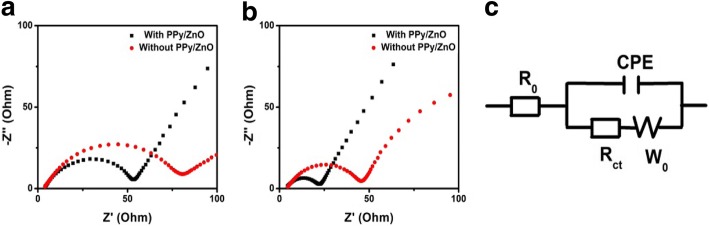
**a–b** EIS plots of the cells with and without PPy/ZnO interlayer before cycle and after 50 cycles and **c** the equivalent circuit

We performed EIS measurements to further investigate the effect of the PPy/ZnO interlayer on charge transfer (Fig. 6). In the high-frequency region, the intercept on the real axis and a depressed semicircle are ascribed to electrolyte ohmic resistance (*R*_o_) and the charge transfer resistance (*R*_ct_), respectively. The tilted straight line in the low-frequency region is ascribed to Warburg impedance [27]. As shown in Fig. 6a, the *R*_ct_ was reduced from 66.3 Ω to 35.9 Ω after the insertion of PPy/ZnO interlayer, which could be because the three-dimensional network of PPy/ZnO interlayer provides faster charge transfer [28]. Even after 50 cycles, the *R*_ct_ for the cell with PPy/ZnO interlayer (12 Ω) was much smaller than that without PPy/ZnO interlayer (33.4 Ω). These results suggest that the PPy/ZnO interlayer not only increases the utilization of active materials but also accelerates fast charge collection/transport [29]. Meanwhile, the difference of Warburg impedance in Fig. 6 was attributed to the fact that the ZnO nanoparticles act positively rather than hinder the diffusion of ions [30].

**Fig. 7:**
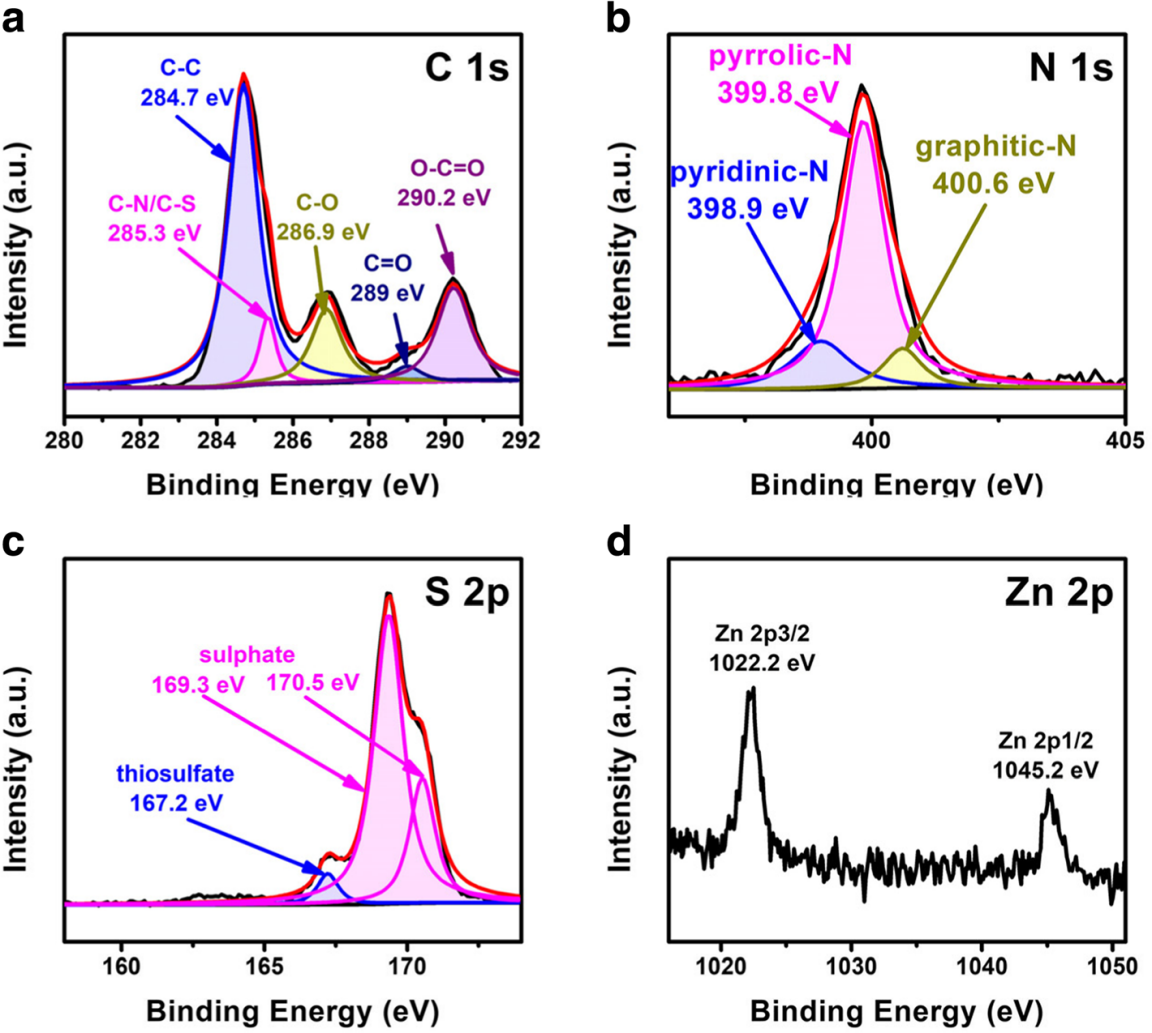
XPS spectra of C 1 s (**a**), N 1 s (**b**), S 2p (**c**), and Zn2p (**d**)

To further investigate the role of PPy/ZnO interlayer in capturing the polysulfides in Li/S batteries, the cell was dismantled after cycling and the bonding configurations of the PPy/ZnO interlayer were studied using C 1 s, N 1 s, S 2p, and Zn 2p XPS spectra (Fig. 7). The samples revealed a strong peak of C-C at about 248.7 eV and multiple peaks between 285 and 292 eV. These multiple peaks correspond to bonds between the hetero atoms or oxygen and carbon, showing the presence of C-N/C-S, C-O, C=O, and O-C=O bonds. As shown in Fig. 7b, there were strong multiple peaks in the range of 398 and 402 eV, namely at 398.9, 399.8, and 400.6 eV, which were ascribed to pyridinic-N, pyrrolic-N, and graphitic-N, respectively. The presence of nitrogen functional groups facilitates the adsorption of the active material during cycling. The sulfur peaks were concentrated in the range of 166 to 172 eV (Fig. 7c). The peak located at 167.2 eV was ascribed to thiosulfate, which is formed due to polysulfide oxidation on the ZnO surface. The other two peaks at around 169.3 to 170.5 eV were attributed to the presence of electrolyte [31]. These results further proved that ZnO nanoparticles can improve the absorption and retention of polysulfides. As presented in the high-resolution Zn 2p XPS spectrum (Fig. 7d), the two peaks centered at 1022.3 and 1045.1 eV are similar to the reported peaks of Zn 2p3/2 and ZnO 2p1/2 [32]. Hence, PPy/ZnO interlayer can absorb and limit polysulfides owing to the strong interaction between PPy/ZnO and polysulfides, which can effectively relax the shuttle effect in Li/S batteries.

## Conclusions

A unique interlayer consisting of a three-dimensional hierarchical network PPy uniformly covered with ZnO nanoparticles was successfully prepared. The prepared interlayer can minimize polysulfide shuttling and effectively protect Li anode to prolong cycle life and improve rate performance of Li/S batteries. The improved performance can be attributed to the physical and chemical interactions of unique three-dimensional hierarchical network structure, nitrogen functional groups, and ZnO nanoparticles to reutilize the dissolved polysulfides. Hence, these preliminary results demonstrate that PPy/ZnO interlayer is a promising strategy for the development of actual applications of high-performance Li/S batteries.

## Additional file

Additional file 1: **Figure S1.** The cycling performance at 0.2 C of cells with PPy/ZnO interlayer, with PPy and without interlayer. (DOC 695 kb)

## Abbreviations

CV: Cyclic voltammetry
DME: 1,2-Dimethoxyethane
DOL: 1,3-Dioxolane
EIS: Electrochemical impedance spectroscopy
FTIR: Fourier-transform infrared spectroscopy
KOH: Potassium hydroxide
Li/S: Lithium/sulfur
LiTFSI: Lithium bis(trifluoromethanesulfonyl)imide
PPy: Polypyrrole
PVDF: Polyvinylidene fluoride
SEM: Scanning electron microscope
TEM: Transmission electron microscope
XPS: X-ray photoelectron spectroscopy
XRD: X-ray diffraction
ZnO: Zinc oxide

## References

[1] Wang DW, Zeng Q, Zhou G, Yin L, Li F, Cheng HM, Gentle IR, Lu GQM (2013). Carbon–sulfur composites for Li–S batteries: status and prospects. J Mater Chem A.

[2] Lin Z, Liang C (2014). Lithium-sulfur batteries: from liquid to solid cells. J Mater Chem A.

[3] Liu J, Qian T, Wang M, Liu X, Xu N, You Y, Yan C (2017). Molecularly imprinted polymer enables high-efficiency recognition and trapping lithium polysulfides for stable lithium sulfur battery. Nano Lett.

[4] Lei TY, Chen W, Huang JW, Yan CY, Sun HX, Wang C, Zhang WL, Li YR, Xiong J (2016). Multi-functional layered WS_2_ nanosheets for enhancing the performance of lithium-sulfur batteries. Adv Energy Mater.

[5] Lei TY, Xie YM, Wang XF, Miao SY, Xiong J, Yan CL (2017). TiO_2_ feather duster as effective polysulfides restrictor for enhanced electrochemical kinetics in lithium-sulfur batteries. Small.

[6] Yu XW, Manthiram A (2017). Electrode-electrolyte interfaces in lithium-sulfur batteries with liquid or inorganic solid electrolytes. Acc Chem Res.

[7] Chen W, Lei TY, Wu CY, Deng M, Gong CH, Hu K, Ma YC, Dai LP, Lv WQ, He WD (2018). Designing safe electrolyte systems for a high-stability lithium-sulfur battery. Adv Energy Mater.

[8] Chen W, Qian T, Xiong J, Xu N, Liu X, Liu J, Zhou J, Shen X, Yang T, Chen Y (2017). A new type of multifunctional polar binder: toward practical application of high energy lithium sulfur batteries. Adv Mater.

[9] Chen W, Lei TY, Qian T, Lv WQ, He WD, Wu CY, Liu XJ, Liu J, Chen B, Yan CL, Xiong J (2018). A new hydrophilic binder enabling strongly anchoring polysulfides for high-performance sulfur electrodes in lithium-sulfur battery. Adv Energy Mater.

[10] Sun J, Sun Y, Pasta M, Zhou G, Li Y, Liu W, Xiong F, Cui Y (2016). Entrapment of polysulfides by a black-phosphorus-modified separator for lithium-sulfur batteries. Adv Mater.

[11] Su YS, Manthiram A (2012). Lithium-sulphur batteries with a microporous carbon paper as a bifunctional interlayer. Nat Commun.

[12] Liu M, Li Q, Qin X, Liang G, Han W, Zhou D, He YB, Li B, Kang F (2017). Suppressing self-discharge and shuttle effect of lithium-sulfur batteries with V_2_O_5_-decorated carbon nanofiber interlayer. Small.

[13] Ma G, Wen Z, Wang Q, Chen S, Peng P, Jin J, Wu X (2015). Enhanced performance of lithium sulfur battery with self-assembly polypyrrole nanotube film as the functional interlayer. J Power Sources.

[14] Sun F, Wang J, Long D, Qiao W, Ling L, Lv C, Cai R (2013). A high-rate lithium–sulfur battery assisted by nitrogen-enriched mesoporous carbons decorated with ultrafine La_2_O_3_ nanoparticles. J Mater Chem A.

[15] Wan C, Wu W, Wu C, Xu J, Guan L (2014). A layered porous ZrO_2_/RGO composite as sulfur host for lithium-sulfur batteries. RSC Adv.

[16] Zhang G, Wu HB, Song T, Paik U, Lou XW (2014). TiO_2_ hollow spheres composed of highly crystalline nanocrystals exhibit superior lithium storage properties. Angew Chem Int Ed.

[17] Yu MP, Wang AL, Tian FY, Song HQ, Wang YS, Li C, Hongd J-D, Shi GQ (2015). Dual-protection of a graphene-sulfur composite by a compact graphene skin and an atomic layer deposited oxide coating for a lithium-sulfur battery. Nanoscale.

[18] Yin F, Liu X, Zhang Y, Zhao Y, Menbayeva A, Bakenov Z, Wang X (2017). Well-dispersed sulfur anchored on interconnected polypyrrole nanofiber network as high performance cathode for lithium-sulfur batteries. Solid State Sci.

[19] Vishnuvardhan TK, Kulkarni VR, Basavaraja C, Raghavendra SC (2006). Synthesis, characterization and A.C. conductivity of polypyrrole/Y_2_O_3_ composites. Bull Mater Sci.

[20] Fu Y, Manthiram A (2012). Orthorhombicbipyramidal sulfur coated with polypyrrole nanolayers as a cathode material for lithium–sulfur batteries. J Phys Chem C.

[21] Zhou G, Yin LC, Wang DW, Li L, Pei S, Gentle IR, Li F, Cheng HM (2013). Fibrous hybrid of graphene and sulfur nanocrystals for high-performance lithium-sulfur batteries. ACS Nano.

[22] Yin YX, Xin S, Guo YG, Wan LJ (2013). Lithium-sulfur batteries: electrochemistry, materials, and prospects. Angew Chem Int Ed.

[23] Xiao L, Cao Y, Xiao J, Schwenzer B, Engelhard MH, Saraf LV, Nie Z, Exarhos GJ, Liu J (2012). A soft approach to encapsulate sulfur: polyaniline nanotubes for lithium-sulfur batteries with long cycle life. Adv Mater.

[24] Mukkabla R, Meduri P, Deepa M, Shivaprasad SM, Ghosal P (2017). Sulfur enriched carbon nanotubols with a poly (3,4-ethylenedioxypyrrole) coating as cathodes for long-lasting Li-S batteries. J Power Sources.

[25] Li S, Ren G, Hoque MNF, Dong Z, Warzywoda J, Fan Z (2017). Carbonized cellulose paper as an effective interlayer in lithium-sulfur batteries. Appl Surf Sci.

[26] Ma G, Wen Z, Jin J, Wu M, Wu X, Zhang J (2014). Enhanced cycle performance of Li–S battery with a polypyrrole functional interlayer. J Power Sources.

[27] Yang K, Zhong L, Guan R, Xiao M, Han D, Wang S, Meng Y (2018). Carbon felt interlayer derived from rice paper and its synergistic encapsulation of polysulfides for lithium-sulfur batteries. Appl Surf Sci.

[28] Zhang Z, Wang G, Lai Y, Li J (2016). A freestanding hollow carbon nanofiber/reduced grapheneoxide interlayer for high-performance lithium–sulfur batteries. J Alloy Compd.

[29] Wang J, Yang Y, Kang F (2015). Porous carbon nanofiber paper as an effective interlayer for high-performance lithium-sulfur batteries. Electrochim Acta.

[30] Tang H, Yao SS, Xue SK, Liu MQ, Chen LL, Jing MX, Shen XQ, Li TB, Xiao KS, Qin SB (2018). In-situ synthesis of carbon@Ti_4_O_7_ non-woven fabric as a multi-functional interlayer for excellent lithium-sulfur battery. Electrochim Acta.

[31] Sun W, Ou X, Yue X, Yang Y, Wang Z, Rooney D, Sun K (2016). A simply effective double-coating cathode with MnO_2_ nanosheets/graphene as functionalized interlayer for high performance lithium-sulfur batteries. Electrochim Acta.

[32] Li H, Wei Y, Zhang Y, Zhang C, Wang G, Zhao Y, Yin F, Bakenov Z (2016). In situ sol-gel synthesis of ultrafine ZnOnanocrystals anchored on graphene as anode material for lithium-ion batteries. Ceram Int.

