# Supplementary material for: A PPy/ZnO functional interlayer to enhance electrochemical performance of lithium/sulfur batteries
Source: Nanoscale Res Lett. 2018 Oct 3;13:307. doi: 10.1186/s11671-018-2724-x (PMC6170249; doi:10.1186/s11671-018-2724-x)
Supplement: Supplementary file 1 — Figure S1. The cycling performance at 0.2 C of cells with PPy/ZnO interlayer, with PPy and without interlayer. (DOC 695 kb) [file 11671_2018_2724_MOESM1_ESM.doc]

**Additional file**

The figure (S.1.) shows a comparison of the cycle performance of three different types of batteries.


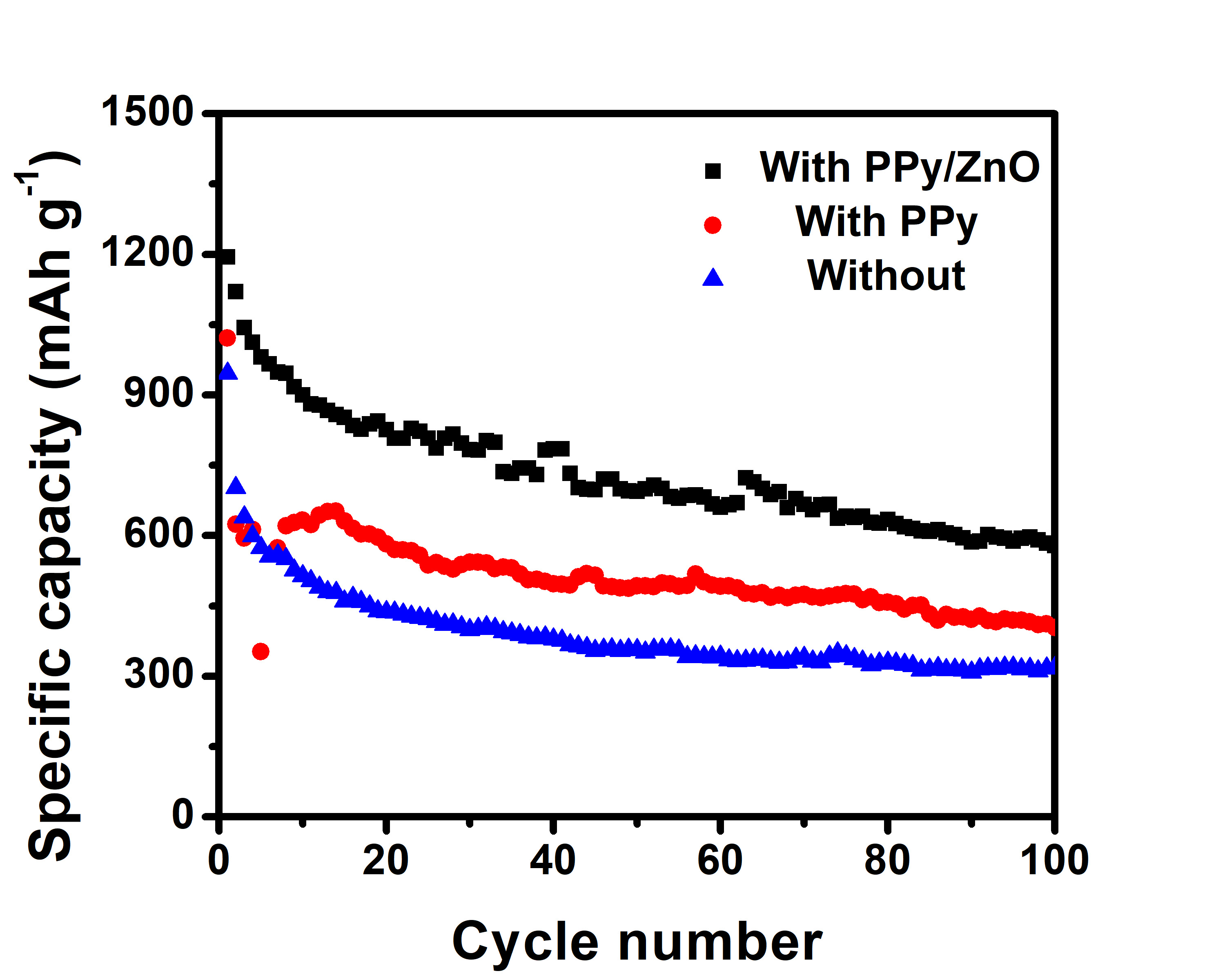


S.1. The cycling performance at 0.2 C of cells with PPy/ZnO interlayer, with PPy and without interlayer.
